# A proposed clinical pathway for the patients with Borderline Personality Disorder presenting to Emergency Departments

**DOI:** 10.1177/10398562231167976

**Published:** 2023-04-05

**Authors:** Beatrice Webb, Jeffrey CL Looi, Stephen Allison, Michael Nance, Rohan Dhillon, Tarun Bastiampillai

**Affiliations:** College of Medicine and Public Health, 1065 Flinders University, Adelaide, SA, Australia; Academic Unit of Psychiatry and Addiction Medicine, 2219 The Australian National University, School of Medicine and Psychology, Canberra Hospital, Canberra, ACT, Australia; Consortium of Australian-Academic Psychiatrists for Independent Policy and Research Analysis (CAPIPRA), Canberra, ACT, Australia; College of Medicine and Public Health, 1065 Flinders University, Adelaide, SA, Australia; Consortium of Australian-Academic Psychiatrists for Independent Policy and Research Analysis (CAPIPRA), Canberra, ACT, Australia; Margaret Tobin Centre, 14351 Flinders Medical Centre, SA, Australia; College of Medicine and Public Health, 8703 Flinders University, Adelaide, SA, Australia; College of Medicine and Public Health, 1065 Flinders University, Adelaide, SA, Australia; Department of Psychiatry, Monash University, Clayton, VIC, Australia; Consortium of Australian-Academic Psychiatrists for Independent Policy Research and Analysis (CAPIPRA), Canberra, ACT, Australia

**Keywords:** borderline personality disorder, emergency department, clinical pathway

## Abstract

**Objective:**

Emergency Department (ED) care of repeated self-injury, intensive affective lability, and interpersonal dysfunction associated with borderline personality disorder (BPD) is challenging. We propose an evidence-based acute clinical pathway for people with BPD.

**Conclusion:**

Our standardised evidence-based short-term acute hospital treatment pathway includes structured ED assessment, structured short-term hospital admission when clinically indicated, and immediate short-term (4-sessions) clinical follow-up. This approach could be adopted nationally to reduce iatrogenic harm, acute service overdependence and negative healthcare system impacts of BPD.

Personality disorders have profound effects on prognosis and treatment. Patients with borderline personality disorder (BPD) who present repeatedly to Emergency Departments (EDs) with self-injury, intense affective lability and interpersonal dysfunction often receive unstructured, reactive, high-intensity and protracted inpatient care, aimed at providing immediate distress containment, despite the lack of evidence for its effectiveness.^1,2,3^ Interpersonal dysfunction associated with BPD increases negative attitudes among clinicians.^4^ Attitudes towards patients with BPD can be negative when compared to patients with other psychiatric diagnoses, partially related to a lack of education and training.^4,5^ This can contribute to and perpetuate stigma, leading to iatrogenic harms such as volatile interactions with ED staff and increasing patient distress.^4^ Lack of evidence-based hospital care may predispose repeat presentations and subsequent dependence on hospital admission for temporary symptom relief.^5^

Structured protocols for BPD in community care have been shown to help clinicians and patients, decreasing iatrogenesis through a reduction in unfocused exploratory and supportive interventions.^6^ However, there is no equivalent consensus on a structured protocol for psychiatric and non-psychiatric staff on the short-term hospital and post-ED care of BPD that adequately addresses all the acute needs of these patients. NHMRC Guidelines sought to improve clinical care for BPD nationwide,^7^ but these recommendations did not fully specify the model of acute care service delivery.^8^ Promising programs exist in some regions,^9^ but these are not available nationwide. A similar structured care pathway for providing rapid-access therapeutic follow-up to patients discharged from the ED following psychosocial crisis involving anxiety and depressive symptomatology has been implemented in South Australia. However, this was designed for patients lacking significant personality pathology and does not address all of the unique challenges faced by those suffering BPD.^10^

We discuss a practical evidence-based acute treatment protocol for those with BPD flowing through EDs, to reduce iatrogenic harm and systemic impacts, aimed at long-term recovery. Our protocol might also reduce costs, through shorter, more-effective admissions.

## BPD related crisis and self-harm

For those suffering BPD, intra- and inter-personal crises are common, and effective treatment can be pivotal.^11^ Due to overwhelming or intense emotions associated with interpersonal conflict or trauma, many people with BPD will engage in suicidal or para-suicidal acts resulting in acute hospital presentations.^12^ 75 percent of people with BPD engage in self-harm and, among these, 90% repeatedly self-harm.^13^ Most of these behaviours are not suicidal and are referred to as non-suicidal self-injury (NSSI).^14^ The number of young Australians presenting to the ED with self-harm has recently risen, some may have BPD.^15^

Suicidal ideas for people with BPD vary in intensity over time, waxing in crisis, and waning during stability.^14^ Patients with BPD have an average of three lifetime suicide attempts.^14^ Suicide attempts vary in intent and severity, and may result in hospital attendance.^14^ Whilst such behaviours do not usually lead to completed suicide, they must be taken seriously by acknowledging and responding appropriately to patients’ distress.^14^

## Crisis access by patients with BPD

Patients with BPD in crisis are often admitted for acute inpatient care, due to a relative shortage of structured follow-up.^11^ However, hospitalisation does not reduce suicide for patients with BPD.^14^ There is a lack of evidence-based protocols for inpatient management of BPD, and the procedure for crisis intervention is often subjective and clinician-dependent.^3,16^ Whether or not to admit patients with BPD may be partly determined by an individual clinician’s fear of litigation, which at times may drive unnecessary involuntary admissions.^14^ Unnecessary admissions may cause harm through psychological trauma and disconnection from ongoing psychotherapeutic approaches.^3^

Repeated, frequent presentations to hospital can lead to negative outcomes including increased suicidality, through interference with out-patient treatment, workplace absences, reduction of self-agency, reinforcement of institutionalised dependency, and potentially, behavioural reinforcement of suicidality.^11,14^ Hospital presentations may be correlated with BPD severity, or may be indicative of the relative insufficiency of existing services to coordinate care in meeting the complex needs of these patients.^17^

Hospital admissions for BPD need to be minimised in number and length-of-stay. Although admissions may be perceived by patients and clinicians as helpful, they may not be therapeutic.^13^

## Acute management of BPD crises—hospitalisation guidelines

**Figure 1.**
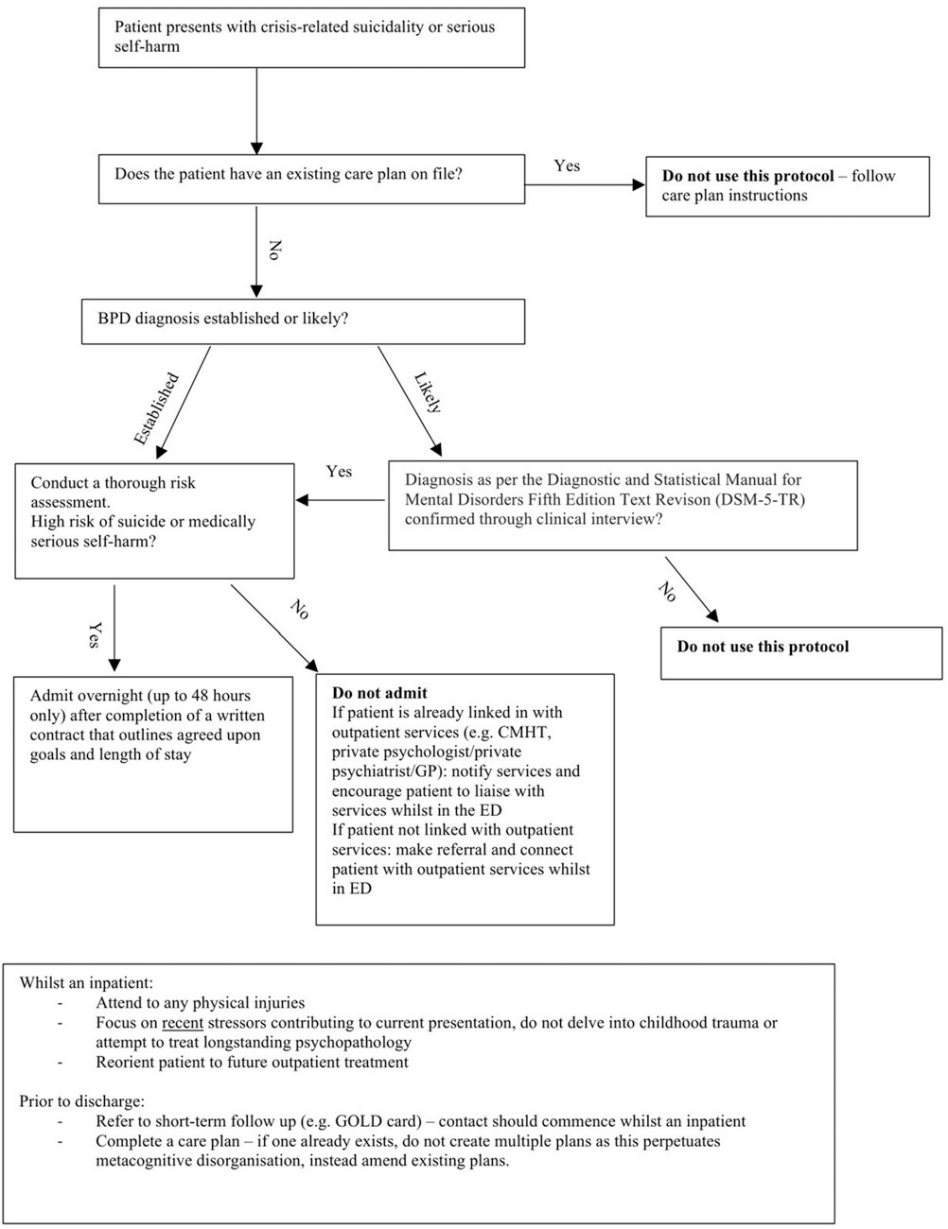
A proposed hospital treatment pathway for patients with BPD in the ED in crisis.

Our proposed care pathway is shown in Figure 1. Patients should be referred to a crisis team or treating clinician as an alternative to admission. Admissions should be short-term, for a higher than baseline suicide risk, re-evaluation after a near-fatal attempt, or a micro-psychotic episode.^7,14^

Intensive assessments that cause iatrogenic harm through exploring developmental trauma should be avoided.^13^ During assessment, focus should remain on the present*:* identifying and mitigating recent stressors, validating strong emotions, emphasising hopefulness for change, and formulating requirements and purpose for admission.^12,13^ An assessment that establishes rapport can be therapeutic and assist in self-regulation.^18^ Existing care plans, which foster autonomy and patient self-determination, should be used to maintain continuity of care.^7^

Admissions should be voluntary, structured, brief (overnight or up to 48 h), and to a specialised emergency psychiatric facility.^11,12,19,20^ Involuntary admissions should be avoided, as they cause iatrogenic deterioration through reinforcing maladaptive behaviours and reduced autonomy.^11,12^

Psychiatric emergency care centres reduce levels of patient aggression, medication use, and the number of patients requiring admission to long-stay psychiatric units.^20^ Admissions should be aimed at achieving specific documented goals that are collaboratively planned with the patient and/or carers,^12,19^ and specify an agreed discharge date.^11^

Patients should be asked what help they are seeking, to enhance their sense of agency and discourage maladaptive self-harming behaviours.^13^ Whilst collaboratively assessing and managing self-harm risk, aims should be to assist the patient to stabilise their mental state, regain control and prepare for future treatment.^12^ Communication and validation will develop rapport, whilst encouraging the patient to find alternative, adaptive, solutions to crises.^16^ Each staff member’s role within the hospital unit and the structured BPD program should be clarified, and the patient should have one primary treating clinician.^12,13^

Clear operational standards for successful BPD management are summarised in Table 1.^19^ The Project Air Strategy for Personality Disorders developed treatment guidelines aimed at improving the capacity of mainstream mental health services to care for people with BPD, also summarised in Table 1.^12^

**Table 1.** Recommendations regarding hospitalisation as per Project Air Guidelines,^12^ and Helleman et al^19^. 2018

Recommended	To be avoided
• Respond to the medical and physical needs of the person first. Provide clear explanations about medical treatment	• Discussing issues related to childhood, relationships or other life situations • Paying a lot of attention to the injury itself, as this will only serve to reinforce self-harming behaviours—find a balance • Involuntary admissions, as it is best to have the person’s consent including agreement on the goals of the admission which should be outlined in a care plan
• When attending to physical injuries or wounds, refrain from reinforcing self- harming behaviours by paying too much attention to it (eg beyond the minimum amount required to stabilise the person). Instead, focus on the triggers that led to the crisis and the course of events from that led to the deliberate self-harm	
• Conduct a thorough risk assessment	
• Develop a plan for admission together with the patient, with clear goals and planned date of discharge	
• Only provide brief admission together with treatment by a community care professional. Once the person’s level of risk has stabilised, determine if the person is currently engaged in longer-term treatment with a health practitioner, or not currently engaged in longer- term treatment	
• If the patient is engaged with longer term treatment, ensure they have a follow up appointment	
• If the patient is not currently engaged with longer term treatment (or there is a significant gap between now and their follow-up appointment), refer the patient for a brief intervention clinic for follow up upon discharge	
• Discharge after the person’s level of risk has stabilised AND they have been referred for a follow-up appointment with a brief intervention clinic or primary treating clinician	

Individual patient care plans should be created prior to discharge, and updated on subsequent admissions.^7^ These can reduce risk of harm and frequency of crisis, ensuring consistency, sense of agency and collaborative care.^12^ Elements may include a daily maintenance plan, triggers, early warning signs and a specific action plan for future crises.^12^

## Brief-intervention clinics post-ED crisis presentations

Brief and rapid psychological intervention supports those with BPD at risk of significant harm.^9^ Brief-intervention clinics have been shown to reduce distress, reduce suicidal ideation, reduce service utilisation, and improve quality of life.^12,21^ Some Australian brief-intervention clinics are located within an acute community-based mental health team closely linked with emergency and inpatient services. An example is the ‘Gold Card’ service, developed by the Project Air Strategy for Personality disorders, components of which are shown in Table 2.^9^

**Table 2.** Components of the Gold Card service.^9^

*Referrals*	• Referrals are made following a crisis presentation at an emergency Department of psychiatric inpatient unit
	• Can be made by: Emergency Departments, psychiatric emergency care Centre/Short-Stay units, community mental health teams
*Appointments*	• 4 appointments offered within 1–3 days of referral
*Aims*	• Provide a timely and rapid response to people seeking treatment in crisis
	• Provide an alternative to hospitalisation or facilitate early discharge
	• Provide brief interventions to help manage the client’s immediate needs
	• Provide brief clinical services aimed at helping the client solve their problems
	• Provide assessment and psycho-education to help the client understand their problems
	• Provide tools and strategies to help the client prevent and better manage future crises
	• Provide an opportunity to assess the client’s needs, including the possible need for other services where necessary
	• Provide an opportunity to connect with the person’s family, partner or carer where desirable
	• Provide treatments with an evidence-base that are effective with personality disorders

Patients should be seen by brief-intervention clinicians whilst the patient is an inpatient, or within 72 h of ED presentation.^12^ The time-limited nature (4-sessions) of these interventions should be emphasised to the patient from initial engagement, to avoid perceived abandonment. The patient should be encouraged to access ongoing evidence-based psychotherapeutic outpatient care after the brief-intervention, facilitated via clinical handover.

## Limitations

Our protocol may be somewhat challenging to implement as a nationwide strategy. Short-stay psychiatric units and brief-intervention follow-up clinics may not exist, therefore staffing, in the context of existing shortages, may prove challenging. There is also a risk that if patients are not admitted for NSSI, they may engage in escalating behaviour in order to have their needs met, requiring a considered clinical response. Finally, highly structured approaches to BPD, while necessary,^11^ may paradoxically lessen personalised care.

## Conclusion

Effective collaborative care for people with BPD can be tailored to the person’s needs, guided by a structured pathway.^7^ We propose: ED assessment and discharge if possible; short-term admission if necessary (overnight or up to 48 h); structured follow-up (4- sessions); and linkage back to longer-term outpatient care. Coherence, consistency, and continuity of treatment all optimise care for people with BPD,^4^ reducing unnecessary clinical care variation.
